# Multi-Planar VMAT Plans for High-Grade Glioma and Glioblastoma Targeting the Hypothalamic-Pituitary Axis Sparing

**DOI:** 10.3390/life12020195

**Published:** 2022-01-28

**Authors:** Eva Y. W. Cheung, Shirley S. H. Ng, Sapphire H. Y. Yung, Dominic Y. T. Cheng, Fandy Y. C. Chan, Janice K. Y. Cheng

**Affiliations:** 1 School of Medical Health and Sciences, Tung Wah College, 19/F, 31 Wylie Road, Ho Man Tin, Hong Kong, China; 17002595@twc.edu.hk (S.H.Y.Y.); 18001627@twc.edu.hk (D.Y.T.C.); 17003980@twc.edu.hk (F.Y.C.C.); 17002895@twc.edu.hk (J.K.Y.C.); 2 Department of Clinical Oncology, Queen Mary Hospital, Pok Fu Lam, Hong Kong, China; nsh420a@ha.org.hk

**Keywords:** high-grade glioma, glioblastoma, volumetric modulated arc therapy, multi-planar VMAT, dual planar VMAT, hypothalamus, pituitary, optic chiasm, hippocampus

## Abstract

Background: This study aimed to identify the better arc configuration of volumetric modulated arc therapy (VMAT) for high-grade glioma and glioblastoma, focusing on a dose reduction to the hypothalamic–pituitary axis through an analysis of dose-volumetric parameters, as well as a correlation analysis between the planned target volume (PTV) to organs at risk (OAR) distance and the radiation dose. Method: Twenty-four patients with 9 high-grade glioma and 15 glioblastomas were included in this study. Identical CT, MRI and structure sets of each patient were used for coplanar VMAT (CO-VMAT), dual planar VMAT (DP-VMAT) and multi-planar VMAT (MP-VMAT) planning. The dose constraints adhered to the RTOG0825 and RTOG9006 protocols. The dose-volumetric parameters of each plan were collected for statistical analysis. Correlation analyses were performed between radiation dose and PTV-OARs distance. Results: The DP-VMAT and MP-VMAT achieved a significant dose reduction to most nearby OARs when compared to CO-VMAT, without compromising the dose to PTV, plan homogeneity and conformity. For centrally located OARs, including the hypothalamus, pituitary, brain stem and optic chiasm, the dose reductions ranged from 2.65 Gy to 3.91 Gy (*p* < 0.001) in DP-VMAT and from 2.57 Gy to 4 Gy (*p* < 0.001) in MP-VMAT. Similar dose reduction effects were achieved for contralaterally located OARs, including the hippocampus, optic nerve, lens and retina, ranging from 1.06 Gy to 4.37 Gy in DP-VMAT and from 0.54 Gy to 3.39 Gy in MP-VMAT. For ipsilaterally located OARs, DP-VMAT achieved a significant dose reduction of 1.75 Gy to D_max_ for the optic nerve. In the correlation analysis, DP-VMAT and MP-VMAT showed significant dose reductions to centrally located OARs when the PTV-OAR distance was less than 4 cm. In particular, DP-VMAT offered better sparing to the optic chiasm when it was located less than 2 cm from the PTV than that of MP-VMAT and CO-VMAT. DP-VMAT and MP-VMAT also showed better sparing to the contralateral hippocampus and retina when they were located 3–8 cm from the PTV. Conclusion: The proposed DP-VMAT and MP-VMAT demonstrated significant dose reductions to centrally located and contralateral OARs and maintained the high plan qualities to PTV with good homogeneity and conformity when compared to CO-VMAT for high-grade glioma and glioblastoma. The benefit in choosing DP-VMAT and MP-VMAT over CO-VMAT was substantial when the PTV was located near the hypothalamus, pituitary, optic chiasm, contralateral hippocampus and contralateral retina.

## 1. Advances in Knowledge

In this study, dual planar volumetric modulated arc therapy (DP-VMAT) and multi planar VMAT (MP-VMAT) demonstrated high plan quality, good dose conformity and homogeneity in PTV, with favourable dose reductions to centrally located and contralateral organs at risk (OARs) when compared to coplanar VMAT (CO-VMAT). The benefit in choosing DP-VMAT and MP-VMAT over CO-VMAT was substantial when the planned target volume (PTV) was located near the hypothalamus, pituitary, optic chiasm, contralateral hippocampus and contralateral retina. 

## 2. Introduction

The incidences of high-grade glioma and glioblastoma are increasing in all age groups globally in the recent decade [1,2]. Surgery, chemotherapy and radiotherapy play an important role in local tumour control, which is crucial in improving patient survival and prolongs their lives [3]. The long-term side effects, including cognitive, visual and auditory impairment and endocrine dysfunction, are of major concern after multi-modality treatments [4,5].

With respect to the infiltrative nature of high-grade glioma and glioblastoma, a large treatment volume is anticipated for full coverage of a tumour in radical treatment. Radiation beam trajectories have to be chosen carefully to deliver a high radiation dose to the planned target volume (PTV), as well as minimize the radiation dose to the nearby organs at risk (OARs). Volumetric modulated arc therapy (VMAT) facilitates the conformal dose delivery to the PTV by variation in dose rate and field size aperture with a gantry that rotates at varied speeds. It has been applied for treating prostate cancer [6,7] and liver cancer [8,9] in coplanar VMAT and lung cancer in non-coplanar VMAT [10]. In view of cranial regions, previous studies suggested that non-coplanar VMAT demonstrated a dose reduction to organs at risk (OARs) located in the peripheral regions, including the hippocampus [11,12,13], normal brain tissue, the temporal lobe and the cochlea [14,15]. A significant dose reduction to these organs helped to minimize the side effects, such as cognitive decline and auditory impairment. While for the OARs located centrally, including the hypothalamus, pituitary and optic chiasm, the radiation dose needed to be planned carefully to minimize the risk of endocrine dysfunction and visual deficit. 

Hypothalamic and pituitary dysfunction after radiotherapy have long been discussed [16,17,18,19,20]. Previous study demonstrated that 82% of patients suffered endocrine deficiency, with 69% experiencing hypothalamic disease and 39% experiencing pituitary deficiency after head and neck radiotherapy [21]. Hypothalamic damage can occur, even with a dose less than 40 Gy. As the primary site of damage, insufficient hypothalamic trophic factor secretion may induce pituitary atrophy, which accelerates the hypopituitarism [22]. Previous studies revealed that growth hormone (GH) deficiency may occur when the pituitary gland receives 30 Gy, and thyroid stimulating hormone deficiency may occur if it receives over 40 Gy [17,23,24]. The risk of hypopituitarism increases steadily from 15 Gy to 70 Gy without a particular threshold [23]. To minimize the dose to the hypothalamic–pituitary axis is crucial for patients’ long-term benefit. 

VMAT delivers a concentrated dose to the isocentre through multiple intensity-modulated arcs, and irradiation to normal tissue is inevitable. Modification of the beam entrance through multiple gantry angles and arc trajectories from different planes may help to disperse the dose deposition to the nearby OARs while maintaining the high dose to the PTV with good conformity and homogeneity. In addition, the radiation dose to the nearby OARs was controlled by the dose prescribed to the PTV, as well as the PTV-OAR distance. Different trajectories of a VMAT plan may have varied dose reduction effects with respect to different PTV-OAR distances. 

In this study, we analysed the dose-volumetric parameters of coplanar VMAT (CO-VMAT), dual planar VMAT (DP-VMAT) and multiplanar VMAT (MP-VMAT) and performed correlation analyses between PTV-OAR distance and the dose received by that OAR to identify the better arc configuration of VMAT to achieve dose reduction to the hypothalamic–pituitary axis.

## 3. Materials and Methodology

### 3.1. Patient Selection

Twenty-four patients with high-grade gliomas or glioblastomas, who were treated in the clinical oncology department of Queen Mary Hospital (QMH), Hong Kong West Cluster of Hospital Authority, Hong Kong from 2012 to 2020 were selected retrospectively. Patients who were treated in a supine position with a base plate, a headrest and a thermoplastic cast as immobilization devices were eligible for this study. Patients who were treated with cranio-spinal irradiation; were diagnosed with brain metastases, carcinomatous meningitis, leptomeningeal diseases; had spinal cord compression; or had PTV overlapping with the brainstem, hypothalamus or pituitary gland were excluded. 

### 3.2. Image Collection and Processing

Simulator CT images, MRI and CT structure sets of each patient were anonymized and collected from QMH. All images were co-registered and were used to delineate the clinical target volume (CTV), PTV and OARs, including the hypothalamus, pituitary gland, brainstem, optic chiasm, optic nerves, lens and retinae. The CTV and PTV were contoured by the oncologists in QMH. The OARs were contoured by the radiation therapists or dosimetrists in the QMH. The hippocampus and the hypothalamus were contoured by the investigators of this study, with references to the previous protocols [24].

### 3.3. Ethics Approval

Ethics application was submitted to and has been approved by the Institutional Review Board of the University of Hong Kong/Hospital Authority Hong Kong West Cluster (HKU/HA HKW IRB No: UW20-866).

### 3.4. Treatment Design and Planning

All treatments plans were planned on a Varian True Beam (Varian Medical System, Palo Alto, California) linear accelerator using Eclipse Radiotherapy Treatment Planning System (Varian Medical Systems, Palo Alto, CA, USA), version 15.6. The dose prescription was 2 Gy per fraction (2 Gy/fr) at the PTV daily for 30 fractions to a total of 60 Gy. Photon beam energy of 6 MV was used. with a maximum dose rate of 600 monitor units (MU) per minute, and gantry speed was set at 4.8° per second. The field sizes were customized using a high-definition multileaf collimator (HD-MLC) 120 with sliding window and were custom fitted in Eclipse Treatment Planning System using the arc geometry tool. The collimator rotation was set at 30° and 330° for clockwise and anti-clockwise arcs, respectively, to minimize inter-leaf leakage. All plan optimizations were carried out in Eclipse Treatment Planning System, with the progressive resolution optimizer (PRO3, v.15.6, Varian Medical systems, Palo Alto, CA, USA). 

**Figure 1:**
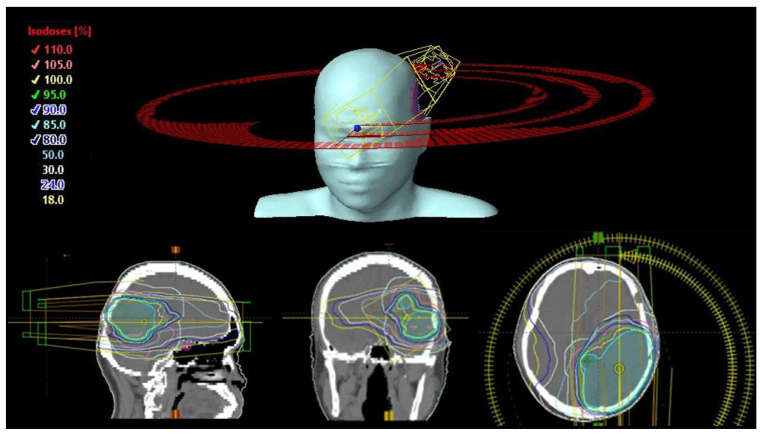
CO-VMAT Arc configuration. Couch rotation was kept at 0° for all arcs.

**Figure 2:**
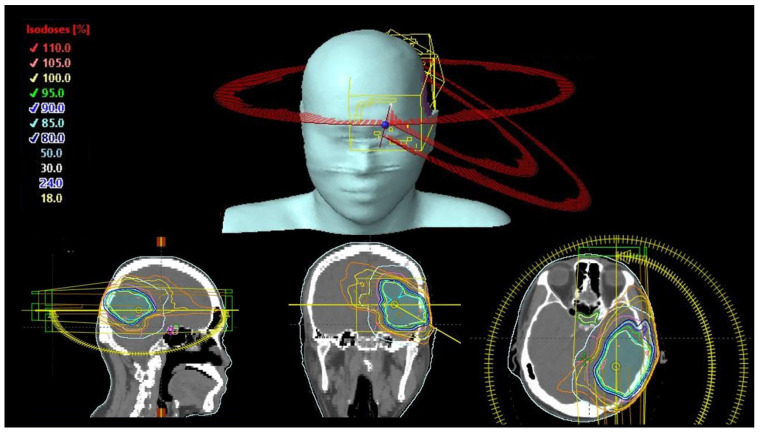
DP-VMAT Arc and couch configuration. Couch rotation was kept at 0° for the full arc and at 30° from inferior for the two half arcs.

**Figure 3:**
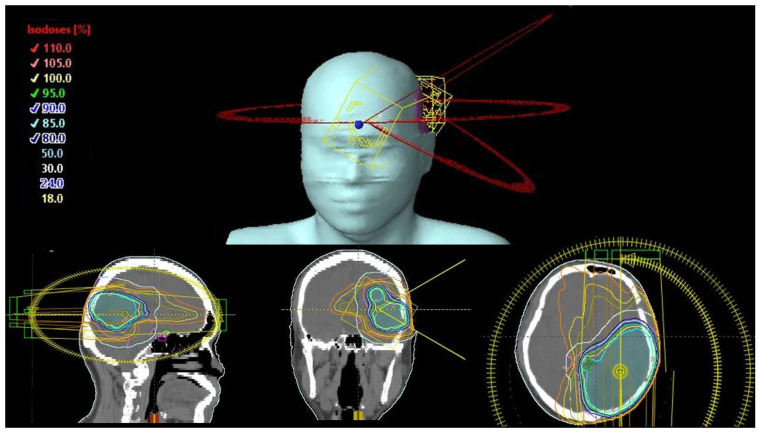
MP-VMAT Arc and couch configuration. Couch rotation was kept at 0° for the full arc, at 30° from superior for one half arc and 30° from inferior for another half arc.

For each patient, coplanar VMAT (CO-VMAT), dual-planar VMAT (DP-VMAT) and multi-planar VMAT (MP-VMAT) plans were generated. For all plans, there was 1 full arc with gantry angle of 179–181° and 2 half arcs with gantry angle of 0° to 179° and 179° to 0° when the PTV was located in the left hemisphere of the brain or 0° to 181°and 181°to 0° when the PTV was located in the right hemisphere of the brain. Couch rotation for all arcs in the CO-VMAT plan and for the full arc for both DP-VMAT and MP-VMAT were set at 0°. While for the half arcs, the couch rotation were set at 330° and 30° in both half arcs for PTV located at the left and right hemisphere of the brain, respectively, in DP-VMAT. For MP-VMAT, couch rotation was set at 330° in one half arc and 30° for another half arc. The arc and couch configuration of CO-VMAT, DP-VMAT and MP-VMAT are shown in Figure 1, Figure 2 and Figure 3, respectively.

A couch rotation of 30° was employed in both DP-VMAT and MP-VMAT. The plans were simulated in the linear accelerator to ensure that no beam went through the patients’ shoulders nor collide with the patients or the immobilization devices. 

### 3.5. Inter-Planner Variability

All treatment plans were created by four investigators who received VMAT optimization training. To minimize the plan variabilities between the planners, three sets of images from the patients were randomly selected as samples for all investigators to plan CO-VMAT, DP-VMAT and MP-VMAT. The dose-volumetric parameters of each plan were analysed to assess the inter-planner variabilities [25]. All four investigators met the specific planning goals and attained similar plan qualities before plan creation for the current study.

### 3.6. Treatment Plan Optimization

All plans were optimized based on the RTOG0825 [26] and RTOG 9006 [27] protocols, fulfilling all dose constraints for critical organs or OARS listed in Table 1 and Table 2. With respect to the PTV, 98% of the volume (D_98%_) received more than 60 Gy, which is 100% of the prescribed dose, while 2% of the volume receive not more than 64.8 Gy, which is 108% of the prescribed dose. The maximum point dose outside the PTV was kept below 64.8 Gy. Both the dose constraints and weighting for the hypothalamus, pituitary and cochlea were not applied. This was to ensure that the dose reduction effects achieved in this study were solely due to the arc configurations and not due to the optimizer.

After optimization, the Anisotropic Analytical Algorithm (AAA, v15.6, Varian Medical systems, Palo Alto, CA, USA) in the Eclipse system was used for dose calculation with a grid size of 1.25 mm. All plans were checked and approved by at least one certified medical dosimetrist prior to the collection of plan qualities and dose-volumetric parameters. 

### 3.7. Plan Qualities and Dose-Volumetric Parameters

The plan qualities were assessed by a homogeneity index and conformation number, which are standard tools to evaluate radiotherapy plans. The homogeneity index was calculated as the dose received by 2% of the PTV (D_2%_) minus the dose received by 98% of the PTV (D_98%_) divided by 60 Gy (prescribed dose to PTV) [28].

HI = (D_2%_ − D_98%_)/prescribed dose (1)

The conformation number was calculated by multiplying the following two factors: (1) the volume of the PTV receiving 60 Gy (prescribed dose), i.e., V_tpr_ divided by total volume of PTV, i.e., VT. (2) The volume of the PTV receiving 60 Gy (prescribed dose), i.e., V_tpr_ divided by the volume enclosed by the isodose of 60 Gy, i.e., V_pr_ [29].

CI = (V_tpr_/VT) × (V_tpr_/V_pr_) (2)

For the PTVs, the maximum dose (D_max_), minimum dose (D_min_), mean dose (D_mean_), minimum dose to 98% of volume of PTV (D_98%_), D_95%_, D_50%_ and D_2%_ were calculated. The total monitor units (MU) of each plan were collected.

For centrally located OARs, i.e., the hypothalamus, pituitary, brainstem and optic chiasm, the D_max_ and D_mean_ of each organ were calculated. For peripherally located OARs, i.e., the optic nerve, lens and retina, the D_max_ and D_mean_ of both the ipsilateral and contralateral organ were calculated. For the hippocampus, D_max_, D_mean_ and D_40%_ were calculated. The normal brain volume received 30 Gy (V_30 Gy_) and 15 Gy (V_15 Gy_) was also calculated. 

The nearest points between the PTV and the OAR were measured from the CT image and recorded as the PTV-OAR distance.

### 3.8. Statistical Analysis

A non-parametric Wilcoxon signed-rank test was used to analyse the differences for each dose-volumetric (DV) parameter among the three plans. *p* values of less than 0.05 were considered to be statistically significant differences. 

A scatterplot was used to illustrate the correlation between the PTV-OAR distance and the dose received (D_max_ and D_mean_) by the corresponding OAR. A linear trend line was plotted to indicate the dose-distance relationship for each plan. All statistical tests were performed using the IBM Statistical Package for the Social Sciences (SPSS) (Version 23.0).

## 4. Results

### 4.1. Patient Demographics

Twenty-four patients fit the eligibility criteria and were included in this study. The male-to-female ratio was 15:9, with ages ranging from 28 to 77. Nine patients were diagnosed as high-grade glioma, and fifteen patients were diagnosed as glioblastoma. All patients received surgery prior to radiotherapy. The PTV volume ranged from 97.6 cm^3^ to 581 cm^3^.

### 4.2. Dose-Volumetric Parameters of the PTV and Total Monitor Units

The PTV dose-volumetric parameters are listed in Table 3 for all three plans. The overall dose-volumetric parameters of all three plans were good and achieved all dose constraints (D_98%_ < 60 Gy, D_2%_ < 64.8 Gy and D_max_ outside PTV < 64.8 Gy) with no significant differences. 

The mean MU of CO-VMAT, DP-VMAT and MP-VMAT were 626.6 ± 107.86, 643.86 ± 93.48 and 585.18 ± 78.05, respectively.

### 4.3. Centrally Located OAR Dose-Volumetric Parameters 

The centrally located OAR dose-volumetric parameters are listed in Table 4. The overall dose-volumetric parameters of the hypothalamus, pituitary, optic chiasm and brainstem of DP-VMAT and MP-VMAT were significantly lower than those of CO-VMAT. The D_max_ and D_mean_ in all above-mentioned organs were 2.54 Gy to 3.75 Gy lower in DP-VMAT than that in CO-VMAT. MP-VMAT exhibited similar results as DP-VAMT, with a significant dose reduction of 2.57 Gy to 3.51 Gy when compared to CO-VAMT, except that the mean dose in the brainstem in MP-VMAT was 0.46 Gy, which was not statistically significant.

### 4.4. Dose-Volumetric Parameters of Ipsilaterally Located OARs

The dose-volumetric parameters of OARs located in the ipsilateral side of the PTV are shown in Table 5. The dose-volumetric parameters of the hippocampus, lens and retina in DP-VMAT and MP-VMAT were similar to those in CO-VMAT. While DP-VMAT demonstrated a significant dose reduction of 1.75 Gy in D_max_ and 0.81 Gy in D_mean_ to the ipsilateral optic nerve when compared to CO-VMAT.

### 4.5. Dose-Volumetric Parameters of Contralaterally Located OARs 

The dose-volumetric parameters of OARs located on the contralateral side of the PTV are shown in Table 6. Similar to the centrally located OARs, DP-VMAT and MP-VMAT demonstrated significant dose reductions to the contralateral hippocampus, optic nerve and retina, ranging from 1.06 Gy to 4.37 Gy dose reductions by DP-VMAT, and 0.54 Gy to 3.39 Gy dose reductions by MP-VMAT when compared to CO-VMAT, except that the dose reduction to the contralateral lens was not statistically significant in MP-VMAT when compared to CO-VMAT. 

### 4.6. Percentage of Normal Brain Tissue Volume Receiving a Low Dose (V30Gy or V15Gy)

The normal brain tissue that received 30 Gy and 15 Gy in CO-VMAT, DP-VMAT and MP-VMAT are listed in Table 7. In DP-VMAT, the percentage of volume reduction in V30Gy and V15Gy were 1.89% and 5.22%, respectively, when compared to CO-VMAT. For MP-VMAT, the percentage of volume reduction in V30Gy and V15Gy were 2.03% and 4.4%, respectively, when compared to CO-VMAT.

### 4.7. Correlation Analysis for PTV-OAR Distance and the Dose Received by the Corresponding OAR

1. The correlation between the D_max_ or D_mean_ of centrally located organs and their distance to the PTV

**Figure 4:**
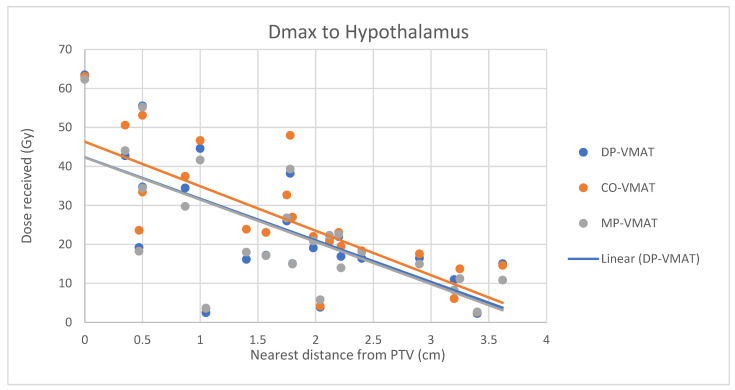
Correlation between the Dmax to the hypothalamus and its distance to the PTV.

**Figure 5:**
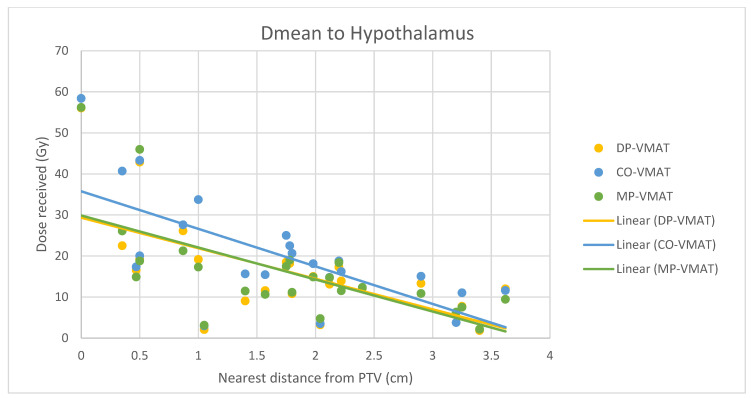
Correlation between the Dmean to the hypothalamus and its distance to the PTV.

**Figure 6:**
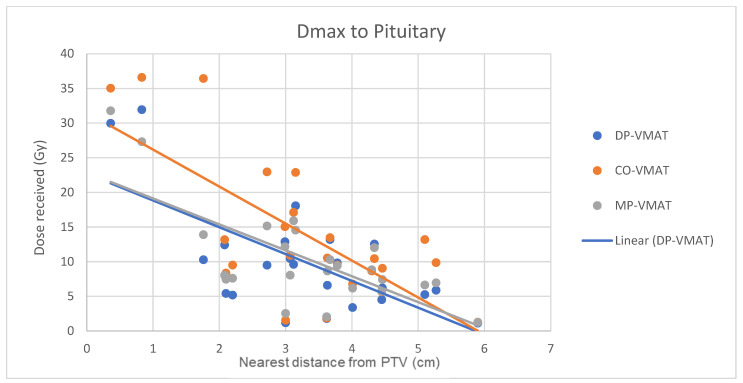
Correlation between the Dmax to the pituitary and its distance to the PTV.

**Figure 7:**
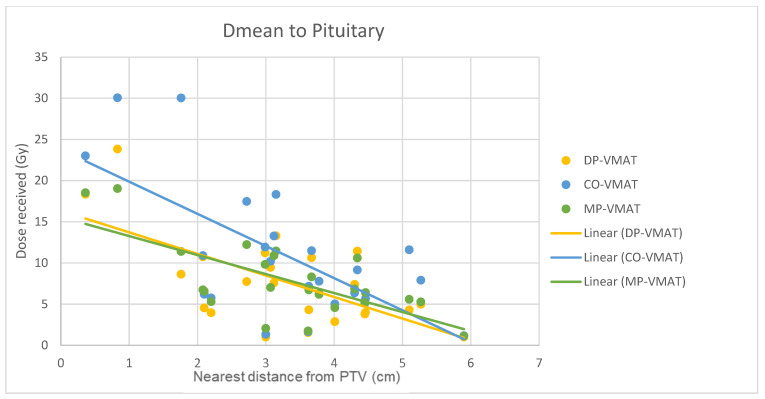
Correlation between the Dmean to the pituitary and its distance to the PTV.

**Figure 8:**
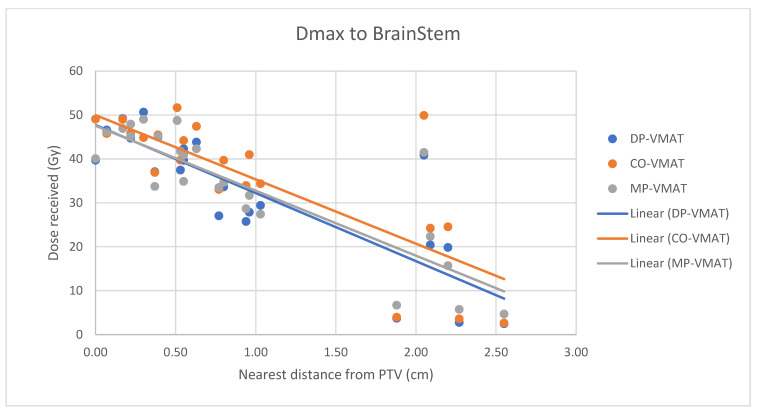
Correlation between the Dmax to the brainstem and its distance to the PTV.

**Figure 9:**
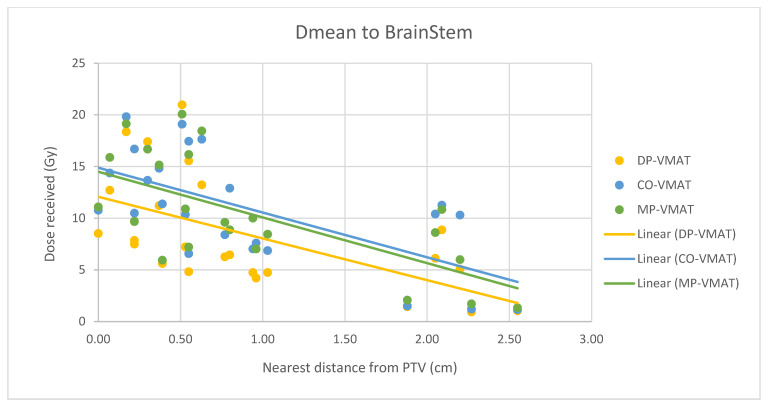
Correlation between the Dmean to the brainstem and its distance to the PTV.

**Figure 10:**
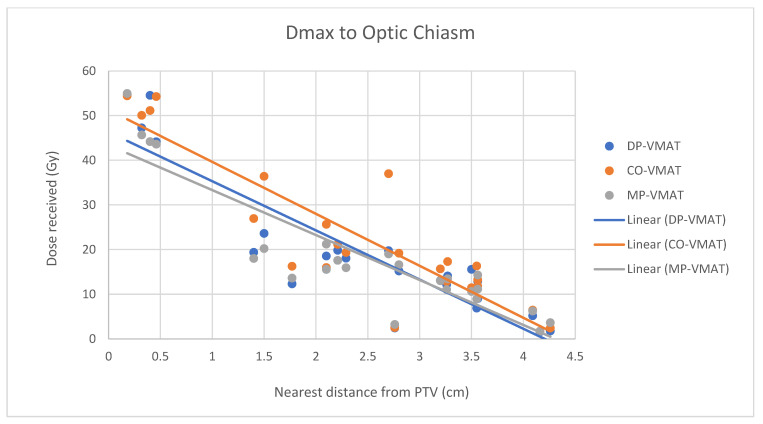
Correlation between the Dmax to the optic chiasm and its distance to the PTV.

**Figure 11:**
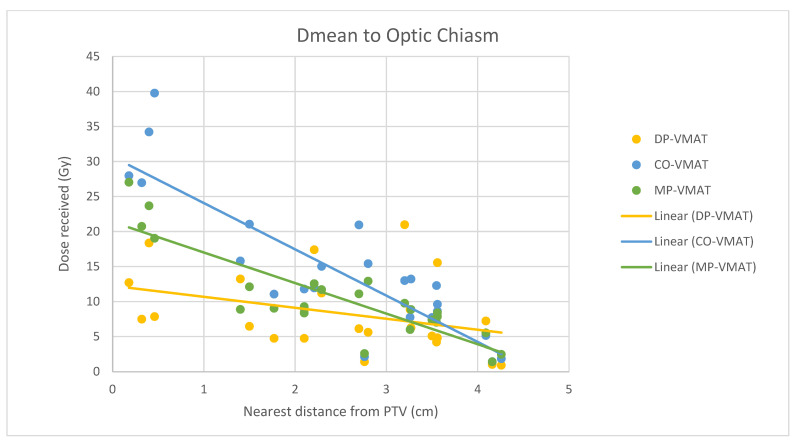
Correlation between the Dmean to the optic chiasm and its distance to the PTV.

For centrally located organs, both the D_max_ and D_mean_ were significantly reduced using DP-VMAT or MP-VMAT if the organ was located within 4 cm from the PTV, when compared to CO-VAMT. For the D_max_ or D_mean_ of the pituitary, using DP-VMAT and MP-VMAT the dose was reduced by 5–8 Gy if it was located less than 2 cm from PTV, when compared to CO-VMAT. For the D_mean_ of the optic chiasm, DP-VMAT and MP-VMAT reduced the dose by 15 Gy and 9 Gy, respectively, if it was located less than 1 cm from PTV. For the brainstem, DP-VMAT and MP-VMAT reduced the dose by 2 Gy when compared to CO-VMAT, regardless of its location from the PTV. The results suggested that DP-VMAT and MP-VMAT are better options than CO-VMAT when the OAR concern is near to the PTV. The results were presented in the Figure 4, Figure 5, Figure 6, Figure 7, Figure 8, Figure 9, Figure 10 and Figure 11.

2. Correlation between the D_max_ or D_mean_ of the peripherally located organs and their distance to the PTV

For ipsilateral OARs, there was no significant correlation between the D_max_ or D_mean_ of peripherally located organs and their distance to the PTV. 

**Figure 12:**
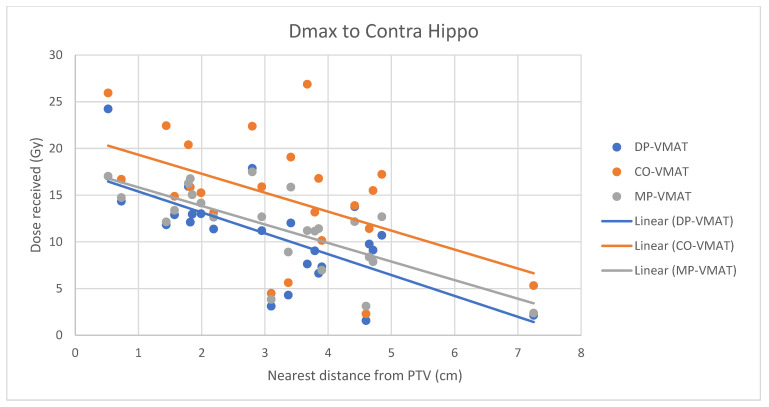
Correlation between the Dmax to the contralateral hippocampus and its distance to the PTV.

**Figure 13:**
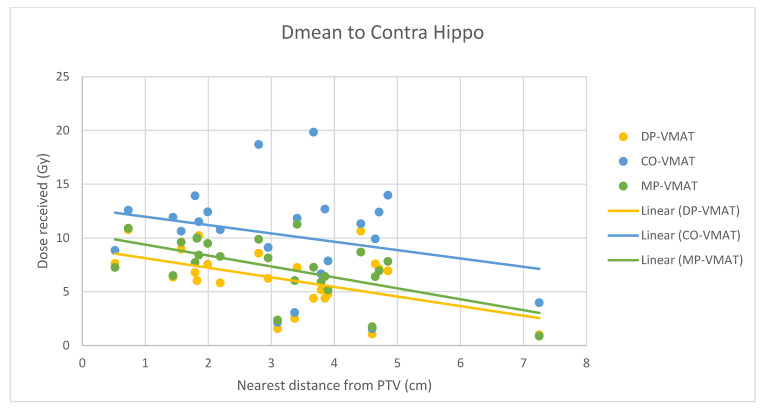
Correlation between the Dmean to the contralateral hippocampus and its distance to the PTV.

**Figure 14:**
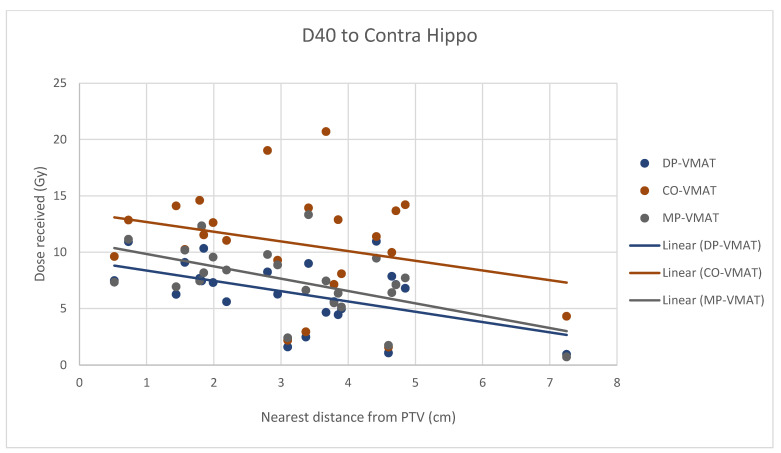
Correlation between the D40 to the contralateral hippocampus and its distance to the PTV.

**Figure 15:**
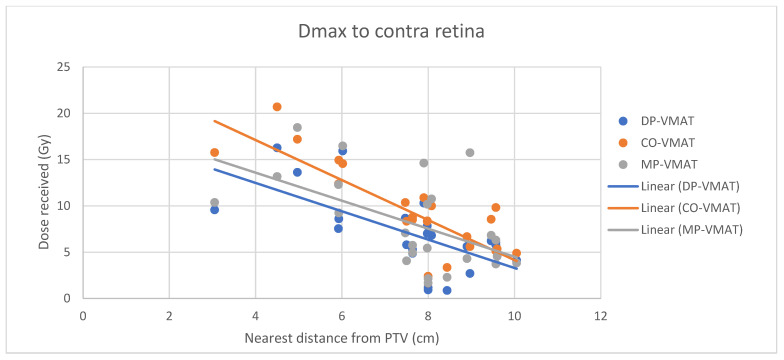
Correlation between the Dmax to the contralateral retina and its distance to the PTV.

**Figure 16:**
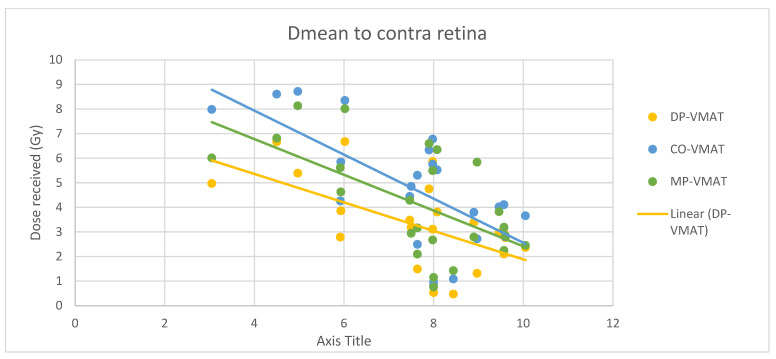
Correlation between the Dmean to the contralateral retina and its distance to the PTV.

However, for the D_max_ and D_mean_ of the contralateral hippocampus, MP-VMAT offered a dose reduction of 3 Gy when compared to CO-VMAT, while DP-VMAT reduced the does by 4 Gy from that of CO-VMAT, regardless of the distance from the PTV. For the D_max_ and D_mean_ of the contralateral retina, DP-VMAT and MP-VMAT offered more dose reduction than CO-VMAT when it was located 3–6 cm from the PTV. The results are presented in Figure 12, Figure 13, Figure 14, Figure 15 and Figure 16.

## 5. Discussion

### 5.1. Significance of the Study

In this study, DP-VMAT and MP-VMAT demonstrated high plan quality, good dose conformity and homogeneity in PTV, with favourable dose reductions to centrally located and contralaterally located OARs when compared to CO-VMAT. The benefit in choosing DP-VMAT and MP-VMAT over CO-VMAT was substantial when the PTV was located near the hypothalamus, pituitary, optic chiasm, contralateral hippocampus and contralateral retina. 

### 5.2. Hypothalamus and Pituitary Sparing

To minimize the long-term complications due to growth hormone (GH), adrenocorticotropic hormone (ACTH), thyroid stimulating hormone (TSH), luteinizing hormone (LH) and follicle stimulating hormone (FSH) insufficiency, it is suggested to minimize the irradiation to the hypothalamic–pituitary axis to less than 30 Gy [19,23]. In our study, the D_max_ to the hypothalamus delivered from 3 VMAT plans were under 30 Gy, while DP-VMAT and MP-VMAT demonstrated better sparing with 11.1% and 12.7% dose reductions, respectively, when compared to CO-VMAT. In the correlation analysis, the dose reduction effect was substantial when the PTV was located less than 2 cm from the hypothalamus and less than 4 cm from the pituitary. A previous study showed that the risk of hypopituitarism was related to the D_mean_ of the pituitary without a clear cut off threshold, with an increased risk starting from 15 Gy [23]. In the correlation analysis, the D_mean_ to the pituitary of DP-VMAT and MP-VMAT were 15 Gy, while it was 23 Gy in CO-VMAT when the pituitary was less 0.3 cm from PTV. Our results suggest that DP-VMAT and MP-VMAT demonstrated a significant sparing effect to the pituitary, especially when it was located near the PTV.

### 5.3. Optic Chiasm, Retinae and Lens Sparing

Radiation-induced optic neuropathy developed when irradiation accumulated to 50 Gy to the optic chiasm, which causes bilateral visual loss, or 10 Gy to the anterior part of optic pathway, which causes unilateral visual loss [30]. With respect to the QUANTEC review, 50 Gy of whole organ dose is associated with a less than 1% risk of blindness. For a dose between 55–60 Gy, the risk of blindness is approximately 3–7%. In this study, all plans were optimized based on the dose constraints from RTOG 0825 and RTOG9006, which restricted the dose to optic chiasm to 56 Gy, optic nerve to 55 Gy, retinae to 50 Gy and lens to 7 Gy [26,27]. The D_max_ and D_mean_ to all organs of optic pathway were well under the normal tissue tolerance for standard fractionation [31]. Late complications of visual loss were not anticipated.

### 5.4. Hippocampus Sparing

It was challenging to spare the hippocampus when radical radiotherapy was prescribed with a standard dose of 60 Gy to the PTV, which usually involved a large brain volume in glioblastoma. Due to the proximity of the ipsilateral hippocampus to the PTV (the hippocampus was inside the PTV in 71% of cases in this study), using DP-VMAT or MP-VMAT could not provide a significant dose reduction when compared to CO-VMAT. However, DP-VMAT and MP-VMAT exhibited over 4 Gy and over 3 Gy dose reduction to D_max_, D_mean_ and D_40%_ of the contralateral hippocampus by DP-VAMT and MP-VMAT, respectively. In addition, the dose reduction effect remained the same, regardless of its distance from the PTV. Gondi et al. 2012 revealed that when the D_40%_ of the hippocampus received more than 7.3 Gy, the patient may suffer long-term cognitive impairment [24]. Our results showed that the D_40%_ of the contralateral hippocampus were 10.32 Gy, 6.42 Gy and 7.51 Gy in CO-VMAT, DP-VMAT and MP-VMAT, respectively. Both DP-VMAT and MP-VMAT were under the suggested threshold, but not CO-VMAT. Compared to intensity-modulated radiotherapy (IMRT), which delivered 24.9 Gy to D_mean_ of the contralateral hippocampus [12], our suggested arc configurations in DP-VMAT and MP-VMAT provided better sparing to the contralateral hippocampus when compared to CO-VMAT and IMRT. 

### 5.5. Normal Brain Tissue Sparing

Referring to the QUANTEC review, the risk of radiation-induced necrosis is less than 3% for a dose of less than 60 Gy to normal brain tissues, and the risk increases to 5% for a dose of 72 Gy [32,33]. As the prescription dose in this study was 60 Gy to the PTV, the risk for radionecrosis was not high. Nevertheless, cognitive impairment may occur after the standard treatment, which consists of concomitant chemo irradiation and adjuvant chemotherapy using temozolomide [34,35]. A recent study reported that patients demonstrated cognitive decline in multiple domains, including executive function, memory and verbal learning [35] after they received the standard treatment suggested by RTOG0825. The inclusion of bevacizumab, an inhibitor of vascular endothelial growth factors (VEGF), in the standard chemo irradiation regime resulted in a greater deterioration in neurocognitive function [36]. Irradiation to normal brain tissue can be another contributor to the cognitive impairment, as it may lead to microglial activation decline [37]. To minimize the risk of decline in neurocognitive function, reducing the normal brain tissue volume being irradiated is a more controllable way, when compared to systemic chemotherapy. The results of this study demonstrated that the percentage of normal brain tissue receiving 30 Gy and 15 Gy were reduced by 1.89% and 5.22%, respectively, in DP-VMAT and 2.03% and 4.4%, respectively, in MP-VMAT, when compared to CO-VMAT. Our results were consistent with the Audet et al. study, which indicated that non-coplanar VMAT reduced the normal brain tissue volume receiving a low dose when compared to coplanar VMAT [38]. 

### 5.6. Treatment Safety

Implementing couch rotation in arc therapy may increase the possibility of a collision between the immobilization devices and the gantry head of the linear accelerator. The chance of collision depends on the degree of couch rotation, tumour location, the size of the patient and the size of immobilization devices [39]. In this study, a 30° couch rotation was employed, and it was free from collision when applied to cranial radiotherapy. In clinical practice, all plans with couch rotation were suggested to simulate in the linear accelerator prior to the first treatment to ensure no potential collision. Manual couch rotation is recommended to be employed for each couch rotation during treatment delivery to minimize the risk of couch collision.

### 5.7. Treatment Delivery Time

With an additional couch rotation in DP-VMAT and two rotations in MP-VMAT, it is anticipated that the treatment time will be longer than a 1.8 Gy CO-VMAT cranial radiotherapy, which has been reported as 2 min per fraction [40]. The couch rotation required less than one minute per movement if it moved manually. In this case, DP-VMAT and MP-VMAT required 3 min and 4 min, respectively. The treatment delivery time of DP-VMAT and MP-VMAT remained acceptable when compared to other treatment techniques. 

### 5.8. Limitations

The first limitation is the sample size is small. To improve the significance of the study, we used matched pair samples rather than independent samples. To achieve this, for each patient, we delineated the RT structures based on the co-registered CT and MRI pair individually. Then, the identical image dataset of each patient was used to optimize the three plans. The paired t-test could be used, as the data was in the form of matched pairs. The variance of the DV parameter difference was smaller than that in the case of independent samples. Non-parametric statistical tests were used to test the significance of the small samples [41]. 

To minimize interleaf leakage from the multi-leaf collimator, a collimator angle of either 330° or 30° was set in all arcs for the three plans. A previous study achieved a significant improvement in PTV homogeneity in non-coplanar VMAT when compared to coplanar VMAT [42]. However, a similar high level of PTV homogeneity was achieved in all three plans in this study. The major discrepancy may be due to the collimator settings, where they kept 0° for all arcs. Further investigation could be conducted to identify the contributing factor for the improvement of PTV homogeneity.

Only one machine (Varian TrueBeam linear accelerator), with the jaw tracking technique enabled, was used to design all plans in this study. The machine was chosen with respect to the availability in the clinical site. Previous studies suggested that the jaw tracking technique helped to reduce the dose to critical organs without changing the calculation algorithm [43,44]. Further investigation could be conducted using other linear accelerators to confirm the dose reduction to OARs were solely contributed by the technique difference but not by other planning parameters.

## 6. Conclusions

The proposed DP-VMAT and MP-VMAT achieved good dose coverage and homogeneity to the PTV and significant dose reductions to the OARs located centrally and those located at the contralateral side of tumours, when compared to CO-VMAT. The benefit of using DP-VMAT and MP-VMAT over CO-VMAT was substantial when the PTV was located near the hypothalamus, pituitary, optic chiasm, contralateral hippocampus and contralateral retina.

## Figures and Tables

**Table 1:** Dose constraints for optimization [26,27].

Critical Organ or Organ-at Risk	Dose Constraints
Brainstem	Maximum dose < 60 Gy
Hypothalamus	Maximum dose < 30 Gy
Pituitary gland	Maximum dose < 30 Gy
Optic Chiasm	Maximum dose < 56 Gy
Optic Nerve	Maximum dose < 55 Gy
Retinae	Maximum dose < 50 Gy
Lens	Maximum dose < 7 Gy
Hippocampus	Maximum dose < 17 Gy

**Table 2:** Treatment planning goals.

Structure	Dose Constraint	Weight
PTV	Maximum dose < 66 Gy	100
	Minimum dose < 60 Gy	100
Eye Balls	Maximum dose < 7 Gy	5
	Mean dose < 35 Gy	
Lenses	Maximum dose < 5 Gy	5
Hippocampus	Maximum dose < 11 Gy	5
	D_40%_ < 9 Gy	10
Hypothalamus	N/A	N/A
Pituitary	N/A	N/A
Cochlear	N/A	N/A

**Table 3:** The PTV dose-volumetric parameters.

PTV DV Parameters	CO-VMAT	DP-VMAT	MP-VMAT	DP-VMAT-CO-VMAT		MP-VMAT-CO-VMAT	
	Mean ± S.D.	Mean ± S.D.	Mean ± S.D.	Diff	*p*	Diff	*p*
Maximum Dose (Gy)	65.63 ± 0.90	65.61 ± 0.66	65.05 ± 0.67	−0.02	0.69	−0.56	0.32
Mean Dose (Gy)	61.54 ± 0.26	61.54 ± 0.23	61.50 ± 0.26	0.00	0.82	0.04	0.38
Minimum Dose (Gy)	48.06 ± 3.74	49.75 ± 5.79	49.44 ± 5.66	1.68	0.83	1.38	0.14
D_98%_ (Gy)	60.27 ± 0.55	60.33 ± 0.55	60.27 ± 0.31	0.06	0.14	0.00	0.88
D_95%_ (Gy)	60.75 ± 0.297	60.77 ± 0.27	60.70 ± 0.17	0.03	0.35	−0.05	0.33
D_50%_ (Gy)	61.56 ± 0.308	61.54 ± 0.27	61.51 ± 0.30	−0.02	0.36	0.05	0.23
D_2%_ (Gy)	62.73 ± 0.311	62.80 ± 0.28	62.63 ± 0.39	−0.08	0.40	0.097	0.20
Homogeneity Index	0.0410 ± 0.0117	0.0413 ± 0.01153	0.0394 ± 0.00883	0.0003	0.65	0.0016	0.40
Conformity Index	1.1146 ± 0.083	1.1092 ± 0.07734	1.0996 ± 0.07135	0.0054	0.38	0.015	0.08

**Table 4:** Dose-volumetric parameters of the centrally located OARs.

Centrally Located OARs	DV Parameters	CO-VMAT	DP-VMAT	MP-VMAT	DP-VMAT-CO-VMAT		MP-VMAT-CO-VMAT	
		Mean ± S.D.	Mean ± S.D.	Mean ± S.D.	Diff	*p*	Diff	*p*
Hypothalamus	Max Dose (Gy)	26.18 ± 16.55	23.54 ± 16.05	23.20 ± 15.79	−2.65 *	0.04	−2.98 *	0.003
	Mean Dose (Gy)	19.61 ± 13.5	16.19 ± 12.10	16.09 ± 12.34	−3.42 *	<0.001	−3.51 *	0.001
Pituitary	Max Dose (Gy)	13.74 ± 10.18	9.83 ± 7.75	10.47 ± 7.09	−3.91 *	<0.001	−3.33 *	0.001
	Mean Dose (Gy)	10.77 ± 7.96	7.62 ± 5.47	7.9 ± 4.50	−3.15 *	<0.001	−2.87 *	0.003
Brain Stem	Max Dose (Gy)	36.54 ± 14.7	33.48 ± 14.72	33.96 ± 13.87	−3.05 *	0.001	−2.57 *	0.013
	Mean Dose (Gy)	10.90 ± 5.33	8.36 ± 5.47	10.44 ± 5.35	−2.54 *	<0.001	−0.46	0.864
Optic Chiasm	Max Dose (Gy)	22.48 ± 16.37	19.07 ± 15.5	18.48 ± 14.19	−3.41 *	0.002	−4.00 *	<0.001
	Mean Dose (Gy)	14.32 ± 9.86	10.57 ± 7.04	10.57 ± 6.39	−3.75 *	<0.001	−3.75 *	<0.001

* *p* value < 0.05.

**Table 5:** Dose-volumetric parameters of the ipsilaterally located OARs.

Ipsilaterally Located OARs	DV Parameters	CO-VMAT	DP-VMAT	MP-VMAT	DP-VMAT-CO-VMAT		MP-VMAT-CO-VMAT	
		Mean ± S.D.	Mean ± S.D.	Mean ± S.D.	Diff	*p*	Diff	*p*
Hippocampus	Max Dose (Gy)	54.24 ± 17.64	54.89 ± 16.57	55.35 ± 15.69	0.66	0.361	1.12	0.162
	Mean Dose (Gy)	41.61 ± 18.94	41.91 ± 12.10	41.88 ± 17.63	0.30	0.511	−0.28	0.53
	D_40%_ (Gy)	45.73 ± 21.02	45.89 ± 19.77	46.27 ± 19.61	0.16	0.864	0.53	0.475
Optic Nerve	Max Dose (Gy)	17.95 ± 16.79	16.20 ± 15.62	16.40 ± 15.43	−1.75 *	0.012	−1.55	0.076
	Mean Dose (Gy)	11.91 ± 10.69	11.10 ± 11.12	11.73 ± 10.97	−0.81 *	0.034	−0.18	0.732
Lens	Max Dose (Gy)	5.43 ± 2.68	4.98 ± 2.24	4.99 ± 1.94	−0.45	0.304	−0.44	0.407
	Mean Dose (Gy)	4.15 ± 2.06	3.79 ± 1.82	3.87 ± 1.42	−0.50	0.376	−0.27	0.493
Retina	Max Dose (Gy)	17.52 ± 11.14	16.82 ± 10.41	16.51 ± 10.15	−0.70	0.153	−1.01	0.23
	Mean Dose (Gy)	6.94 ± 3.84	6.61 ± 3.57	6.815 ± 3.57	0.33	0.137	−0.13	0.79

* *p* value < 0.05.

**Table 6:** Dose-volumetric parameters of the contralaterally located OARs.

Contra-Laterally Located OARs	DV Parameters	CO-VMAT	DP-VMAT	MP-VMAT	DP-VMAT-CO-VMAT		MP-VMAT-CO-VMAT	
		Mean ± S.D.	Mean ± S.D.	Mean ± S.D.	Diff	*p*	Diff	*p*
Hippocampus	Max Dose (Gy)	14.99 ± 6.35	10.62 ± 5.17	11.59 ± 4.35	−4.37 *	<0.001	−3.39 *	<0.001
	Mean Dose (Gy)	10.32 ± 4.54	6.22 ± 2.77	7.21 ± 2.67	−4.10 *	<0.001	−3.11 *	<0.001
	D_40%_ (Gy)	10.84 ± 4.77	6.42 ± 2.86	7.51 ± 3.04	−4.42 *	<0.001	−3.34 *	<0.001
Optic Nerve	Max Dose (Gy)	9.10 ± 6.65	6.33 ± 5.21	7.11 ± 4.59	−2.77 *	<0.001	−1.98 *	0.009
	Mean Dose (Gy)	6.86 ± 4.73	4.49 ± 3.18	5.38 ± 3.26	−2.37 *	<0.001	−1.48 *	0.006
Lens	Max Dose (Gy)	4.30 ± 1.98	3.18 ± 1.65	4.05 ± 1.99	−1.13 *	<0.001	−0.25	0.265
	Mean Dose (Gy)	3.56 ± 1.67	2.50 ± 1.30	3.29 ± 1.53	−1.06 *	<0.001	−0.26	0.284
Retina	Max Dose (Gy)	9.27 ± 4.78	6.96 ± 4.11	8.09 ± 4.93	−2.35 *	<0.001	−1.18 *	0.046
	Mean Dose (Gy)	4.68 ± 2.33	3.25 ± 1.77	4.14 ± 2.15	−1.43 *	<0.001	−0.54 *	0.025

* *p* value <0.05.

**Table 7:** Percentage of normal brain tissue volume receiving a low dose, i.e., 30Gy and 15Gy.

Percentage of Normal Brain Tissue	CO-VMAT	DP-VMAT	MP-VMAT	DP-VMAT-CO-VMAT		MP-VMAT-CO-VMAT	
	Mean ± S.D.	Mean ± S.D.	Mean ± S.D.	Diff	*p*	Diff	*p*
30 Gy (%)	21.55 ± 9.08	19.67 ± 8.49	19.52 ± 8.39	−1.89 *	<0.001	−2.03 *	<0.001
15 Gy (%)	50.45 ± 18.30	45.23 ± 16.12	46.05 ± 18.01	−5.22 *	<0.001	−4.40 *	<0.001

* *p* value < 0.05.

## Data Availability

The clinical and MRI data are not publicly available for patient privacy protection purposes.

## References

[1] Fitzmaurice C., Allen C., Barber R.M., Barregard L., Bhutta Z.A., Brenner H., Dicker D.J., Chimed-Orchir O., Dandona R., Global Burden of Disease Cancer Collaboration (2017). Global, Regional, and National Cancer Incidence, Mortality, Years of Life Lost, Years Lived with Disability, and Disability-Adjusted Life-years for 32 Cancer Groups, 1990 to 2015: A Systematic Analysis for the Global Burden of Disease Study. JAMA Oncol..

[2] GBD 2016 Brain and Other CNS Cancer Collaborators (2019). Global, regional, and national burden of brain and other CNS cancer, 1990–2016: A systematic analysis for the Global Burden of Disease Study 2016. Lancet Neurol..

[3] Stupp R., Hegi M.E., Mason W.P., van den Bent M.J., Taphoorn M.J., Janzer R.C., Ludwin S.K., Allgeier A., Fisher B., Belanger K. (2009). Effects of radiotherapy with concomitant and adjuvant temozolomide versus radiotherapy alone on survival in glioblastoma in a randomised phase III study: 5-year analysis of the EORTC-NCIC trial. Lancet Oncol..

[4] Diaz A.Z., Choi M. (2014). Radiation-Associated Toxicities in the Treatment of High-Grade Gliomas. Semin. Oncol..

[5] Douw L., Klein M., Fagel S.S., van den Heuvel J., Taphoorn M.J., Aaronson N.K., Postma T.J., Vandertop W.P., Mooij J.J., Boerman R.H. (2009). Cognitive and radiological effects of radiotherapy in patients with low-grade glioma: Long-term follow-up. Lancet Neurol..

[6] Høyer M., Muren L.P., Glimelius B. (2015). The evolution of radiotherapy techniques in the management of prostate cancer. Acta Oncol..

[7] Kiljunen T., Kangasmäki A., Aaltonen A., Kairemo K., Partanen K., Joensuu G., Alanko T., Vaalavirta L., Joensuu T. (2015). VMAT technique enables concomitant radiotherapy of prostate cancer and pelvic bone metastases. Acta Oncol..

[8] Miura H., Ozawa S., Kusaba H., Doi Y., Kenjo M., Yamada K., Nagata Y. (2020). Characterization of robust optimization for VMAT plan for liver cancer. Rep. Pract. Oncol. Radiother..

[9] Moon Y.M., Jeon W., Yu T., Bae S.I., Kim J.Y., Kang J.-K., Choi C.W. (2020). Which Is Better for Liver SBRT: Dosimetric Comparison Between DCAT and VMAT for Liver Tumors. Front. Oncol..

[10] Cheung E.Y.W., Kwong V.H.Y., Chan F.Y.C., Cheng D.Y.T., Cheng J.K.Y., Yung S.H.Y., Chan K.T.K., Cheung K.T.Y., Cheung T.S.W., Yiu J.C.L. (2021). Modified VMAT Plans for Locally Advanced Centrally Located Non-Small Cell Lung Cancer (NSCLC). Life.

[11] Awad R., Fogarty G., Hong A., Kelly P., Ng D., Santos D., Haydu L. (2013). Hippocampal avoidance with volumetric modulated arc therapy in melanoma brain metastases–the first Australian experience. Radiat. Oncol..

[12] Pinkham M.B., Bertrand K.C., Olson S., Zarate D., Oram J., Pullar A., Foote M.C. (2014). Hippocampal-sparing radiotherapy: The new standard of care for World Health Organization grade II and III gliomas?. J. Clin. Neurosci..

[13] Lanaspèze C., Varela Cagetti L., Vieillevigne L. (2016). Hippocampal sparing whole brain radiotherapy with volumetric modulated arc therapy. Phys. Med..

[14] Cheung E.Y.W., Lee K.H.Y., Lau W.T.L., Lau A.P.Y., Wat P.Y. (2021). Non-coplanar VMAT plans for postoperative primary brain tumour to reduce dose to hippocampus, temporal lobe and cochlea: A planning study. BJR Open.

[15] Bas Ayata H., Ceylan C., Kılıç A., Güden M., Engin K. (2018). Comparison of Multiple Treatment Planning Techniques for High-Grade Glioma Tumors Near to Critical Organs. Oncol. Res. Treat..

[16] Constine L.S., Woolf P.D., Cann D., Mick G., McCormick K., Raubertas R.F., Rubin P. (1993). Hypothalamic-Pituitary Dysfunction after Radiation for Brain Tumors. N. Engl. J. Med..

[17] Toogood A.A. (2004). Endocrine consequences of brain irradiation. Growth Horm. IGF Res..

[18] Darzy K.H., Shalet S.M. (2003). Radiation-Induced Growth Hormone Deficiency. Horm. Res. Paediatr..

[19] Darzy K.H., Shalet S.M. (2005). Hypopituitarism As a Consequence of Brain Tumours and Radiotherapy. Pituitary.

[20] Darzy K.H. (2009). Radiation-induced hypopituitarism after cancer therapy: Who, how and when to test. Nat. Clin. Pract. Endocrinol. Metab..

[21] Samaan N.A., Vieto R., Schultz P.N., Maor M., Meoz R.T., Sampiere V.A., Cangir A., Ried H.L., Jesse R.H. (1982). Hypothalamic, pituitary and thyroid dysfunction after radiotherapy to the head and neck. Int. J. Radiat. Oncol..

[22] Darzy K.H. (2013). Radiation-induced hypopituitarism. Curr. Opin. Endocrinol. Diabetes Obes..

[23] Partoune E., Virzi M., Vander Veken L., Renard L., Maiter D. (2021). Occurrence of pituitary hormone deficits in relation to both pituitary and hypothalamic doses after radiotherapy for skull base meningioma. Clin. Endocrinol. (Oxf.).

[24] Gondi V., Hermann B.P., Mehta M.P., Tomé W.A. (2012). Hippocampal Dosimetry Predicts Neurocognitive Function Impairment After Fractionated Stereotactic Radiotherapy for Benign or Low-Grade Adult Brain Tumors. Int. J. Radiat. Oncol..

[25] Nelms B.E., Robinson G., Markham J., Velasco K., Boyd S., Narayan S., Wheeler J., Sobczak M.L. (2012). Variation in external beam treatment plan quality: An inter-institutional study of planners and planning systems. Pract. Radiat. Oncol..

[26] Gilbert M.R., Dignam J., Won M., Blumenthal D.T., Vogelbaum M.A., Aldape K.D., Colman H., Chakravarti A., Jeraj R., Armstrong T.S. (2013). RTOG 0825: Phase III double-blind placebo-controlled trial evaluating bevacizumab (Bev) in patients (Pts) with newly diagnosed glioblastoma (GBM). J. Clin. Oncol..

[27] Ali A.N., Zhang P., Yung W.K.A., Chen Y., Movsas B., Urtasun R.C., Jones C.U., Choi K.N., Michalski J.M., Fischbach A.J. (2018). NRG oncology RTOG 9006: A phase III randomized trial of hyperfractionated radiotherapy (RT) and BCNU versus standard RT and BCNU for malignant glioma patients. J. Neurooncol..

[28] Kataria T., Sharma K., Subramani V., Karrthick K., Bisht S. (2012). Homogeneity Index: An objective tool for assessment of conformal radiation treatments. J. Med. Phys..

[29] Van’t Riet A., Mak A.C.A., Moerland M.A., Elders L.H., van der Zee W. (1997). A conformation number to quantify the degree of conformality in brachytherapy and external beam irradiation: Application to the prostate. Int. J. Radiat. Oncol..

[30] Danesh-Meyer H.V. (2008). Radiation-induced optic neuropathy. J. Clin. Neurosci..

[31] Emami B., Lyman J., Brown A., Coia L., Goitein M., Munzenrider J.E., Shank B., Solin L.J., Wesson M. (1991). Tolerance of normal tissue to therapeutic irradiation. Int. J. Radiat. Oncol. Biol. Phys..

[32] Lawrence Y.R., Li X.A., Naqa I., Hahn C.A., Marks L.B., Merchant T.E., Dicker A.P. (2010). Radiation dose-volume effects in the brain. Int. J. Radiat. Oncol. Biol. Phys..

[33] Marks L.B., Yorke E.D., Jackson A., Ten Haken R.K., Constine L.S., Eisbruch A., Bentzen S.M., Nam J., Deasy J.O. (2010). Use of normal tissue complication probability models in the clinic. Int. J. Radiat. Oncol. Biol. Phys..

[34] Stupp R., Mason W.P., van den Bent M.J., Weller M., Fisher B., Taphoorn M.J.B., Belanger K., Brandes A.A., Marosi C., Bogdahn U. (2005). Radiotherapy plus concomitant and adjuvant temozolomide for glioblastoma. N. Engl. J. Med..

[35] Hilverda K., Bosma I., Heimans J.J., Postma T.J., Peter Vandertop W., Slotman B.J., Buter J., Reijneveld J.C., Klein M. (2010). Cognitive functioning in glioblastoma patients during radiotherapy and temozolomide treatment: Initial findings. J. Neurooncol..

[36] Wefel J.S., Armstrong T.S., Pugh S.L., Gilbert M.R., Wendland M.M., Brachman D.G., Roof K.S., Brown P.D., Crocker I.R., Robins H.I. (2021). Neurocognitive, symptom, and health-related quality of life outcomes of a randomized trial of bevacizumab for newly diagnosed glioblastoma (NRG/RTOG 0825). Neuro-Oncology.

[37] Ramanan S., Kooshki M., Zhao W., Hsu F.-C., Riddle D.R., Robbins M.E. (2009). The PPARα Agonist Fenofibrate Preserves Hippocampal Neurogenesis and Inhibits Microglial Activation After Whole-Brain Irradiation. Int. J. Radiat. Oncol..

[38] Audet C., Poffenbarger B.A., Chang P., Jackson P.S., Lundahl R.E., Ryu S.I., Ray G.R. (2011). Evaluation of volumetric modulated arc therapy for cranial radiosurgery using multiple noncoplanar arcs: Volumetric modulated arc therapy for cranial radiosurgery. Med. Phys..

[39] Smyth G., Evans P.M., Bamber J.C., Bedford J.L. (2019). Recent developments in non-coplanar radiotherapy. Br. J. Radiol..

[40] Smyth G., Evans P.M., Bamber J.C., Mandeville H.C., Welsh L.C., Saran F.H., Bedford J.L. (2016). Non-coplanar trajectories to improve organ at risk sparing in volumetric modulated arc therapy for primary brain tumors. Radiother. Oncol..

[41] Xu M., Fralick D., Zheng J.Z., Wang B., Tu X.M., Feng C. (2017). The Differences and Similarities Between Two-Sample T-Test and Paired T-Test. Shanghai Arch. Psychiatry.

[42] Uto M., Mizowaki T., Ogura K., Hiraoka M. (2016). Non-coplanar volumetric-modulated arc therapy (VMAT) for craniopharyngiomas reduces radiation doses to the bilateral hippocampus: A planning study comparing dynamic conformal arc therapy, coplanar VMAT, and non-coplanar VMAT. Radiat. Oncol..

[43] Xie K., Sun H., Gao L., Lin T., Sui J., Ni X. (2019). A comparative study of identical VMAT about two adjacent targets with and without fixed-jaw technique. Radiat. Oncol..

[44] Snyder K.C., Wen N., Huang Y., Kim J., Zhao B., Siddiqui S., Chetty I.J., Ryu S. (2015). Use of jaw tracking in intensity modulated and volumetric modulated arc radiation therapy for spine stereotactic radiosurgery. Pract. Radiat. Oncol..

